# Influence of Liver Fibrosis on Lobular Zonation

**DOI:** 10.3390/cells8121556

**Published:** 2019-12-02

**Authors:** Ahmed Ghallab, Maiju Myllys, Christian H. Holland, Ayham Zaza, Walaa Murad, Reham Hassan, Yasser A. Ahmed, Tahany Abbas, Eman A. Abdelrahim, Kai Markus Schneider, Madlen Matz-Soja, Jörg Reinders, Rolf Gebhardt, Marie-Luise Berres, Maximilian Hatting, Dirk Drasdo, Julio Saez-Rodriguez, Christian Trautwein, Jan G. Hengstler

**Affiliations:** 1 Leibniz Research Centre for Working Environment and Human Factors at the Technical University Dortmund, 44139 Dortmund, Germany, zaza@ifado.de (A.Z.); , Reinders@ifado.de (J.R.); dirk.dras@gmail.com (D.D.); 2 Department of Forensic Medicine and Toxicology, Faculty of Veterinary Medicine, South Valley University, Qena 83523, Egypt; 3 Faculty of Medicine, Institute of Computational Biomedicine, Heidelberg University, Bioquant—Im Neuenheimer Feld 267, 69120 Heidelberg, Germany; christian.holland@bioquant.uni-heidelberg.de (C.H.H.); julio.saez@bioquant.uni-heidelberg.de (J.S.-R.); 4 Faculty of Medicine, Joint Research Centre for Computational Biomedicine (JRC-COMBINE), RWTH Aachen University, Pauwelsstrasse 19, 52074 Aachen, Germany; 5 Histology Department, Faculty of Medicine, South Valley University, Qena 83523, Egypt; walaamurad1991@yahoo.com (W.M.); tahany_abbass@yahoo.com (T.A.); emaneweda@yahoo.com (E.A.A.); 6 Department of Histology, Faculty of Veterinary Medicine, South Valley University, Qena 83523, Egypt; yasser.ali@vet.svu.edu.eg; 7 Department of Medicine III, University Hospital RWTH Aachen, Aachen University, 52074 Aachen, Germany; Kai.Markus.Schneider@gmail.com (K.M.S.); mberres@ukaachen.de (M.-L.B.); mhatting@ukaachen.de (M.H.); ctrautwein@ukaachen.de (C.T.); 8 Faculty of Medicine, Rudolf-Schönheimer-Institute of Biochemistry, Leipzig University, 04103 Leipzig, Germany; Madlen.Matz@medizin.uni-leipzig.de (M.M.-S.); Rolf.Gebhardt@medizin.uni-leipzig.de (R.G.); 9 Modelling and Analysis for Medical and Biological Applications (MAMBA), Inria Paris & Sorbonne Université LJLL, 2 Rue Simone IFF, 75012 Paris, France

**Keywords:** zonation, liver lobule, chronic liver disease, cytochrome P450, inflammation, bile duct ligation, acetaminophen

## Abstract

Little is known about how liver fibrosis influences lobular zonation. To address this question, we used three mouse models of liver fibrosis, repeated CCl_4_ administration for 2, 6 and 12 months to induce pericentral damage, as well as bile duct ligation (21 days) and mdr2^−/−^ mice to study periportal fibrosis. Analyses were performed by RNA-sequencing, immunostaining of zonated proteins and image analysis. RNA-sequencing demonstrated a significant enrichment of pericentral genes among genes downregulated by CCl_4_; vice versa, periportal genes were enriched among the upregulated genes. Immunostaining showed an almost complete loss of pericentral proteins, such as cytochrome P450 enzymes and glutamine synthetase, while periportal proteins, such as arginase 1 and CPS1 became expressed also in pericentral hepatocytes. This pattern of fibrosis-associated ‘periportalization’ was consistently observed in all three mouse models and led to complete resistance to hepatotoxic doses of acetaminophen (200 mg/kg). Characterization of the expression response identified the inflammatory pathways TGFβ, NFκB, TNFα, and transcription factors NFKb1, Stat1, Hif1a, Trp53, and Atf1 among those activated, while estrogen-associated pathways, Hnf4a and Hnf1a, were decreased. In conclusion, liver fibrosis leads to strong alterations of lobular zonation, where the pericentral region adopts periportal features. Beside adverse consequences, periportalization supports adaptation to repeated doses of hepatotoxic compounds.

## 1. Introduction

Prevalence and mortality of liver diseases, including fibrosis and cirrhosis, continue to grow in Europe [1]. The increase in alcohol consumption and obesity-associated non-alcoholic fatty liver disease (NAFLD) in recent years has contributed to this development. Liver fibrosis is a complex wound-healing process that leads to inflammation and scarring [2,3]. It occurs as a consequence of chronic liver damage, caused by different etiologies, including chronic intoxication, viral infections, genetic diseases, or metabolic disorders due to super-nutrition [2]. Liver fibrosis requires the interaction of several cell types, myofibroblasts, macrophages, hepatocytes and immune cells that are orchestrated by a spectrum of cytokines, chemokines and mediators such as lipids, hormones and reactive oxygen species [3,4,5]. Progressive fibrosis is characterized by the excessive accumulation of the extracellular matrix which compromises the functional architecture of the organ [3,6].

Liver zonation is the spatial separation of a large spectrum of different metabolic pathways along the porto-central axis of the liver lobule, which is essential for liver function [7,8]. For example, xenobiotic metabolism by cytochrome P450 enzymes (CYP) is located in the approximately 50% of hepatocytes in the center of the liver lobule [9,10]. This serves to detoxify xenobiotics before they are drained into the central vein [2,11]. However, for compounds metabolically activated by CYPs, such as CCl_4_ or acetaminophen, the zonated expression causes a pericentral pattern of necrosis [12,13]. A zonated metabolic pattern is also known for ammonia metabolism [14]. While urea-cycle enzymes detoxify ammonia by high capacity and low affinity mechanism in the periportal and midzonal regions, low remaining ammonia concentrations are removed from the sinusoidal blood by a pericentral ring of glutamine synthetase positive hepatocytes that act by a low capacity, high affinity mechanism [15,16,17]. Further zonated functions include glycolysis, gluconeogenesis, glycogenesis, the TCA-cycle, glutamine metabolism and lipogenesis [2,7].

While liver fibrosis and the mechanisms of zonation have already been intensively studied [18,19,20,21], little is known about how liver fibrosis influences lobular zonation. In the present study, we used three different mouse models of liver fibrosis, chronic CCl_4_ intoxication, bile duct ligation (BDL) and the knockout of mdr2. For all three fibrosis models, we observed a loss of pericentral factors, while the entire lobule adapts periportal features. Besides of several adverse consequences, ‘periportalization’ is responsible for adaptation to toxic stress during the pathogenesis of liver fibrosis and leads to resistance to the hepatotoxic compound acetaminophen (APAP).

## 2. Materials and Methods

### 2.1. Experimental Animals

Eight to 10 week-old male C57BL/6N mice (Janvier Labs, France) and 8- to 64-week-old Mdr2^−/−^ mice and age-matched controls were used. The mice were housed under 12 h light/dark cycles at a controlled ambient temperature of 25 °C and fed ad libitum on a standard diet (Ssniff, Soest, Germany) with free access to water. Three to 5 mice were used for each time point and condition given in the result section. All experiments were approved by the local animal protection authorities (application number: 84-02.04.2017.A177).

### 2.2. Induction of Chronic Liver Injury by CCl_4_ and Bile Duct Ligation (BDL)

For induction of progressive pericentral fibrosis, carbon tetrachloride (CCl_4_, 1 g/kg b.w. in olive oil) was repeatedly injected intraperitoneally (i.p.) twice a week for 2, 6 and 12 months. The vehicle controls received only olive oil in the same way as the CCl_4_ groups. Samples were collected on day 6 after the last CCl_4_ or oil injection. In order to induce periportal fibrosis, the extrahepatic common bile duct was ligated under anaesthesia as previously described [22]. Liver tissue samples were collected on day 21 post-BDL or sham operation.

### 2.3. Induction of Acute Liver Injury by Acetaminophen (APAP)

In order to induce acute liver injury, a dose of 200 mg/kg b.w. APAP was dissolved in warm phosphate-buffered saline (PBS) and i.p. injected. The mice were fasted overnight before APAP injection.

### 2.4. Sample Collection

At the time points indicated in the result section, the mice were anaesthetised by an i.p. injection of ketamine (100 mg/kg b.w.) and xylazine (10 mg/kg b.w.). After the loss of reflexes, blood as well as liver tissue samples were collected. The blood samples were collected from the portal vein, the hepatic vein, and the right heart chamber in EDTA-coated syringes as previously described [15]. The samples were centrifuged at 13,000 rpm for 10 min in order to separate plasma. The collected plasma was stored at −80 °C until used for analyses. After blood collection, the remaining blood was removed by perfusion trans-cardially with 40 mL PBS. Subsequently, the whole liver was excised and specimens were collected from defined anatomical positions as follow: (i) a specimen of approximately 1 cm size was taken from the left liver lobe, fixed in 4% paraformaldehyde (PFA) for 2 days and then embedded in paraffin for 2D staining; (ii) a specimen of approximately 0.5 cm size was taken from the left liver lobe and immediately embedded in Tissue-Tek® cryomold in Neg-50 media (ThermoFisher Scientific, Oberhausen, Germany), frozen in 2-methylbutane and stored at −80 °C until used for cryosection preparation; (iii) a specimen of approximately 0.5 cm size was taken from the median liver lobe, fixed in 4% PFA for 2 days, incubated in 30% sucrose for 2 days, and then embedded in Tissue-Tek® cryomold in Neg-50 media, frozen in 2-methylbutane and stored at −80 °C until used for preparation of liver slices; (iv) a specimen of approximately 20 mg weight was taken from the left liver lobe, snap-frozen in liquid nitrogen and stored at −80 °C until used for RNA isolation.

### 2.5. Histopathology

Haematoxylin and eosin staining was performed in 5 µm-thick paraffin embedded tissue sections using a standard protocol. In order to visualize collagen accumulation during chronic liver disease progression, picrosirius red staining was performed in 5 µm-thick paraffin embedded tissue sections using a commercially available kit (Polyscience Europe GmbH, Eppelheim, Germany), according to the manufacturer’s instructions.

### 2.6. Gene Expression Analyses

Quantitative real-time polymerase chain reaction (qRT-PCR). Total RNA was isolated from frozen liver tissue using QIAzol Lysis Reagent (Qiagen, Hilden, Germany). cDNA synthesis was performed from 2 µg of isolated RNA using a commercially available kit (High-Capacity cDNA Reverse Transcription Kit, Applied Biosystems, Schwerte, Germany). qRT-PCR analyses were performed using TaqMan 7500 Real-Time PCR, TaqMan universal PCR Master Mix (Applied Biosystems, Schwerte, Germany), and TaqMan gene expression assays (ThermoFisher Scientifics, Oberhausen, Germany). Gene expression values were normalized to GAPDH, calculated using the ΔΔCt method, and are expressed as fold changes over untreated control samples.

### 2.7. RNA-Seq Analysis

Pre-processing and normalization. The count matrix was derived from FASTQ files of wild-type, CCl_4_ plus olive oil and pure olive oil samples using the web application BioJupies [23]. The samples were normalized using the R package edgeR (version 3.25.8) [24].

Differential gene expression analysis. Differential gene expression analysis was performed with the R package limma (version 3.39.18) [25]. To extract the effect of chronic CCl_4_ intoxication, we computed contrasts comparing the treated samples (CCl_4_ plus olive oil) versus matched olive oil samples. While there are matched oil samples for month 2 and 12, there were no oil samples for month 6 available. As the oil effect was relatively constant across time we imputed oil expression values for month 6 by taking the arithmetic mean of month 2 and month 12. A gene was considered as differentially expressed with abs(logFC) ≥ 1.5 and FDR ≤ 0.05.

### 2.8. Functional Genomics Analysis of the CCl_4_ Signature

Pathway analysis with PROGENy. Pathway activity scores were calculated with the functional genomics tools PROGENy [26,27]. While classical pathway analysis methods (e.g., Kyoto Encyclopedia of Genes and Genomes (KEGG) pathway analysis) rely on gene sets containing genes of pathway members PROGENy exploits so called “footprint gene sets” containing not the pathway members but the most responsive genes upon corresponding pathway perturbation. PROGENy was applied on contrast using the moderated t-value as a gene-level statistic.

Transcription factor (TF) analysis with DoRothEA. DoRothEA is a high-quality data resource of TF-target interactions (regulons) [26,28]. Coupling DoRothEA regulons with a statistical method allows to infer TF activity from the expression of its transcriptional targets. We used as statistical method the function viper from the R package viper (version: 1.17.0) [29] that computes for each TF a normalized enrichment score that we consider as TF activity. DoRothEA in combination with viper was applied on contrasts using moderated t-values as a gene-level statistic.

Gene Ontology (GO) term enrichment. We used the R package msigdf (https://github.com/ToledoEM/msigdf) to query GO terms (biological process and molecular functions) from MsigDB. Gene set enrichment analysis was performed on contrasts using the R package fgsea (version 1.10.0) [30] with 100,000 permutations. The moderated t-value was used as a gene-level statistic.

Construction of consensus pericentral and periportal gene sets. Consensus pericentral and periportal gene sets were constructed by integrating pericentral and periportal gene sets from three independent studies [31,32,33]. Gene sets reported by Braeuning et al. [31] were extracted from their corresponding Supplementary Table S1. Saito et al. [33] made their data available for both male and female mice. Since only male mice were used in the here presented work we focused on common (with respect to male and female mice) and male specific pericentral and periportal genes. Halpern et al. [32] do not provide explicit pericentral and periportal gene sets in their supplement; therefore, we generated them systematically starting from their Supplementary Tables S1 and S2. Supplementary Table S1 contains the UMI counts of 1415 hepatocytes/cells. Supplementary Table S2 reports information about the spatial organization of the 1415 cells across the 9 lobule layer (layer 1 is the most pericentral and layer 9 is the most periportal layer). For each cell and lobule layer combination a probability is given that indicates the likelihood that a cell was originally located in the respective layer. We started our analysis pipeline by removing genes that were expressed in less than 15 cells (out of 1415). Subsequently, we normalized the sub-setted count matrix using the R package scran (version 1.11.27) [34]. To assign cells to a specific lobule layer we selected for each cell the layer with the highest probability yielding in a zonation table reporting the spatial distribution of all cells across the lobule. Given this zonation table we identified genes with significant monotonic expression changes across the lobule layer by applying the exact version of the Jonckheere–Terpstra test from the R package clinfun (version 1.0.15, https://cran.r-project.org/web/packages/clinfun/index.html). Monotonically increasing genes (from layer 1–9) were considered as potential periportal and monotonic decreasing as potential pericentral genes. Only genes with an FDR ≤ 0.001 were considered as pericentral and periportal gene set members. As the three independent studies were published within more than a decade we updated all MGI gene symbols to their current alias using the function alias2SymbolTable from the limma package (version: 3.39.18) [25]. The consensus pericentral and periportal gene sets contain only those genes that are reported in at least two studies.

Characterization of the overlap of pericentral/periportal genes and the most responsive genes of CCl_4_ treatment. In this analysis, the overlap of pericentral and periportal genes with the differentially expressed genes after CCl_4_ treatment is characterized with over-representation analysis (ORA). To ensure a reasonable overlap size we relaxed the condition of differentially expressed genes to abs(logFC) ≥ 0.8 and FDR ≤ 0.2. We identified the final overlap gene set (for both zonations: pericentral and periportal) independently of time by taking the union of overlapping genes across all time points (months 2–12). ORA was performed using Fisher’s exact test. The number of background genes has been set to 20,000 as this reflects a typical number of genes in a mouse transcriptome experiment. We tested the following gene sets: GO terms (molecular functions and biological processes), DoRothEA regulon’s, PROGENy’s footprint gene sets, KEGG gene sets. *P*-values were corrected using Benjamini Hochberg correction (false discovery rate, FDR) [35].

### 2.9. Immunohistochemistry

Immunohistochemistry analysis was performed in five µm-thick frozen or formalin-fixed paraffin-embedded liver tissue sections using antibodies against CYP3A (Biotrend, Cologne, Germany), CYP1A, CYP2C (a gift from Dr. R. Wolf, Biochemical Research Centre, University of Dundee, Dundee, UK), CYP2E1, Arginase1 (Sigma-Aldrich Corp., St. Louis, MO, USA), GS (BD Bioscience, Heidelberg, Germany), and CPS1 (Abcam, Cambridge, UK) (Table 1). The following horseradish peroxidase-conjugated secondary antibodies were used: anti-rabbit IgG (Agilent, Santa Clara, CA, USA), anti-mouse IgG (Sigma-Aldrich Corp., St. Louis, MO, USA), and anti-rat IgG (Linaris GmbH, Heidelberg, Germany) (Table 1). In order to visualize the target signal, the tissues were stained with either 3,3′-diaminobenzidine solution (Vector Laboratories, Peterborough, UK) or AEC+ high sensitivity substrate chromogen (Agilent, Santa Clara, CA, USA). The nuclei were visualized by counter-staining with Mayer’s haematoxylin.

### 2.10. Immunostaining of Liver Slices

CYP2E1 immunostaining was done in 200 µm-thick liver slices prepared using a cryostat microtome. After washing, permeabilization and blocking steps the slices were incubated for 3 days with a primary antibody against CYP2E1 (Sigma-Aldrich Corp., St. Louis, MO, USA, 1:50). Subsequently, the slices were incubated for 2 days with CyTM3-conjugated AffiniPure; F(ab’)2 fragment donkey anti-rabbit secondary antibody (Dianova, Hamburg, Germany, 1:100). The nuclei were visualized by counterstaining with 4,6-diamidino-2-phenylindole. In order to allow 3D imaging of the thick slices, the tissue was cleared by successive immersion in 50% (*v*/*v*), 75% (*v*/*v*) and 100% (*v*/*v*) tetrahydrofuran, 15 min each, followed by immersion in di-benzyl ether (Sigma-Aldrich Corp., St. Louis, MO, USA) for at least 10 min. Subsequently, Z-stacks of approximately 120 µm-depth were acquired using a custom-modified inverted LSM MP7 (Zeiss, Jena, Germany) with an LD C-Apochromat 40 × 1.1 water immersion objective.

### 2.11. Image Analyses and 3D Reconstructions

In order to provide an unbiased and quantitative description of histological slices, a problem-specific image analysis pipeline was developed. Depending on the utilized staining and after performing necessary preprocessing, various related features were segmented. These segmentations were verified by domain knowledge experts, serving as a gold standard. Subsequent measurements were then performed before producing the final summary statistic. Both ImageJ [36] and Matlab R2019a were utilized throughout the quantification process. The former was used for exploration and the later for developing dedicated programs. In the case of bright field scans and after obtaining satisfactory segmentation of a CYP-positive area as well as the associated corresponding background, a ratio was recorded. For the 3D reconstruction, individual structures (nuclei, CYP-positive area and the vessels) were also first segmented and verified. The final reconstruction and visualization were prepared using Imaris 9.3 software.

### 2.12. Ammonia Assay

Ammonia concentrations were measured from 20 µL whole blood samples directly after collection. The analysis was done using the Blood Ammonia Meter PocketChem BA PA-4140 (Arkray.inc, Amstelveen, The Netherlands).

### 2.13. Transaminase Activity Assay

Alanine transaminase (ALT) and aspartate transaminase (AST) activities were measured from plasma samples after dilution 1:10 in PBS. The analysis was done at the Central Laboratory Facility at University Hospital RWTH Aachen (Aachen, Germany).

### 2.14. Statistical Analysis

Statistical analysis of the data other than the RNA-seq was done using SPSS software, version 26. An independent samples *t*-test was used. *p* < 0.05 was considered statistically significant.

## 3. Results

### 3.1. RNA-Seq Demonstrates Downregulation of Pericentral and Upregulation of Periportal Genes in Fibrosis

**Figure 1:**
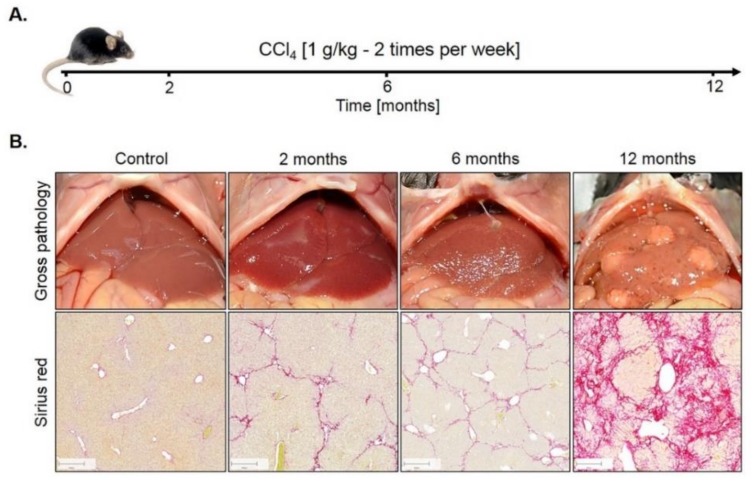
Mouse model of liver fibrosis induced by CCl_4_ administration. (**A**) Treatment schedule. (**B**) Macroscopical alterations and visualization of fibrosis by Sirius red staining. Scale bars: 200 µm.

Genome-wide expression response caused by CCl_4_. To study the influence of fibrosis on liver zonation, we established a mouse model with two intraperitoneal injections of 1 g/kg CCl_4_ per week over 12 months (Figure 1A). Only a relatively mild fibrosis was observed up to six months (Figure 1B). However, between months 6 and 12, the mice progressed into severe fibrosis characterized by wide Sirius red positive fibrotic streets, regenerative nodules and fibrosis-associated macroscopically visible tumor nodules (Figure 1B).

**Figure 2:**
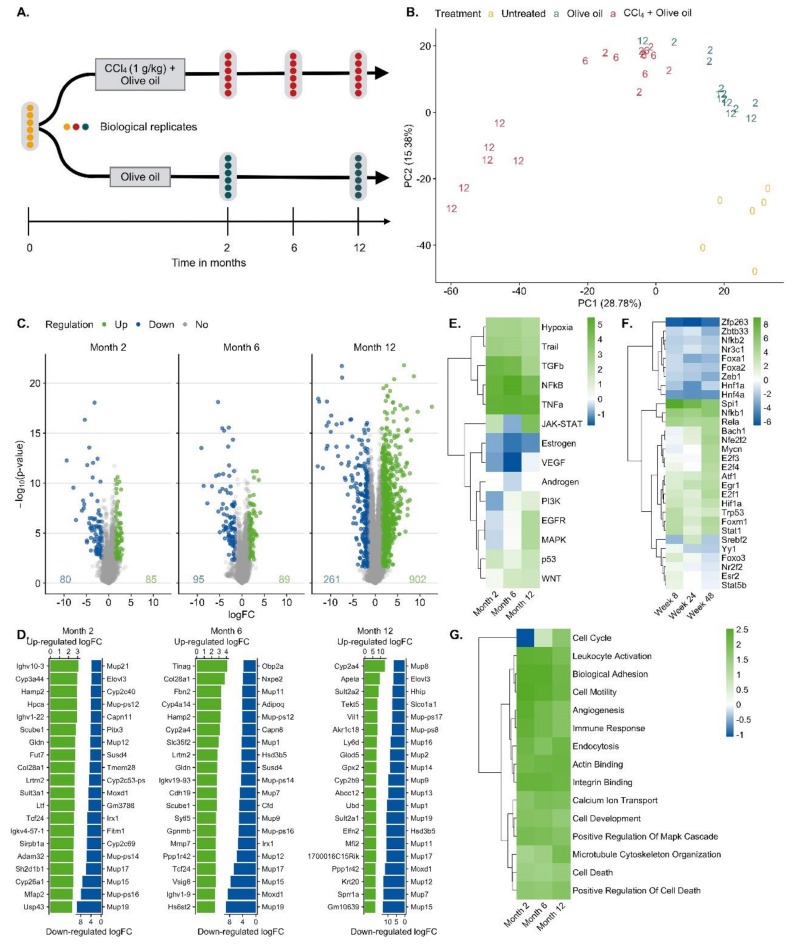
Bioinformatics of RNA-seq data of mouse liver tissue after exposure to CCl_4_ for up to one year. **A**. Analysis schedule; **B**. Principal component analysis (PCA). Untreated mice at the time point zero (0), the day of onset of exposure for the other mouse groups, period of olive oil exposure in blue (2 and 12 months) and period of CCl_4_ (solved in olive oil) exposure (2, 6 and 12 months) in red. **C**. Visualization of significantly up (green) and downregulated (blue) genes after 2, 6 and 12 months of CCl_4_ exposure. **D**. The 20 most up- and downregulated genes after 2, 6 and 12 months exposure to CCl_4_. **E**. Up- and downregulated pathways via PROGENy. The color legend indicates pathway activity (z-score). **F**. Transcription factor (TF) activities computed with DoRothEA. The color legend indicates TF activity (normalized enrichment score, NES). **G**. Enriched Gene Ontology (GO) groups. The color legend indicates the degree of enrichment (NES).

For RNA-seq analysis CCl_4_-treated mice were processed after 0, 2, 6 and 12 months; olive oil controls were included after 2 and 12 months. Liver tissues of six mice per condition were analyzed (Figure 2A). A principal component analysis (PCA) of the RNA-seq data showed a good clustering of each group of six mice (Figure 2B). Treatment with CCl_4_ caused a shift in the inverse direction of principal component 1 (PC1) that explains ~30% of the variance in the data (Figure 2B). PC2 represents the combined effects of olive oil, the solvent of CCl_4_, and aging (Figure 2B). Differential gene expression analysis revealed that 80/85, 95/89 and 261/902 genes were significantly [abs(logFC) ≥ 1.5 and FDR ≤ 0.05] up/downregulated after 2, 6 and 12 months of CCl_4_ treatment compared to olive oil controls, respectively, with partially very strong, more than 1000-fold expression changes (Figure 2C; lists of differential genes: Table S1). Strongly and consistently upregulated genes (Figure 2D) comprise extracellular matrix-associated genes, such as *Col28a1*, whose role in liver fibrosis is well-known; the variable domains of immunoglobulin heavy chains, suggesting infiltration of B cells/plasma cells [37], e.g., Ighv10-3, Ighv1-9, Ighv4-57-1, Ighv1-22, Ighv4-57-1; the liver-derived peptide hormone hepcidin-2 which supports iron homeostasis [38]; and some factors that so far have not been considered as primary genes affected by liver damage, such as gliomedin (*Gldn*), a protein expressed by myelinating Schwann cells [39] and the leucine-rich repeat and transmembrane domain-containing protein 2 (Lrtm2). Among the strongest downregulated genes (Figure 2D) are several major urinary proteins (Mups), also known as α_2_µ-globulins, such as Mup19, Mup21, Mup15, Mup17, Mup-ps16, Mup-ps14 and Mup12. Expression of Mup proteins is known to be induced by androgens and they are physiologically relevant, because they bind small hydrophobic molecules, such as steroid hormones, lipids and retinoids in plasma [40]; several cytochrome P450 enzymes; the sushi domain-containing protein 4 (SUSD4) which inhibits complement factors [41]; calpains (e.g., capn11, Capn8) that act as calcium-dependent cysteine proteases, fatty acid elongase 3 (Elovl3); and roquois homeobox protein 1 (lrx1-6) that is known as a cardiac transcription factor [42]. The role of many of these differential genes in liver fibrosis remains unknown. Characterization of the CCl_4_-induced expression response by pathway analysis using the functional genomics tools PROGENy identified the inflammatory pathways TGFβ, NFκB, TNFα and hypoxia-induced signaling as most active (Figure 2E; Table S2), which is in agreement with previous studies [43]. Estrogen and androgen associated pathways were among the most decreased in the CCl_4_-exposed livers for all time points compared to corresponding oil samples. Transcription factor (TF) activities were inferred with DoRothEA and were dominated by TF with an increased activity that mediate inflammation (e.g., NFKb1, Stat1) cell stress as well as hypoxia response (Hif1a, Trp53, Atf1) and support proliferation (e.g., E2f1, Ef3, Egr1). TFs with reduced activities are known to mediate mature liver functions, such as Hnf4a, Hnf1a, Esr2 and the Fox genes (Figure 2F; Table S3). Enriched GO-terms such as actin-binding, angiogenesis, cell cycle, death and immune response further round out the picture of an inflamed, regenerating tissue (Figure 2G; Table S4).

The solvent controls with olive oil alone showed expression changes after 2 and 12 months, respectively, compared to untreated mice at time zero (Table S5). No age-matched untreated controls were included, because the study was designed to identify CCl_4_ induced expression changes. Administration of CCl_4_ in oil and comparison to oil controls represents a frequently used protocol.

**Figure 3:**
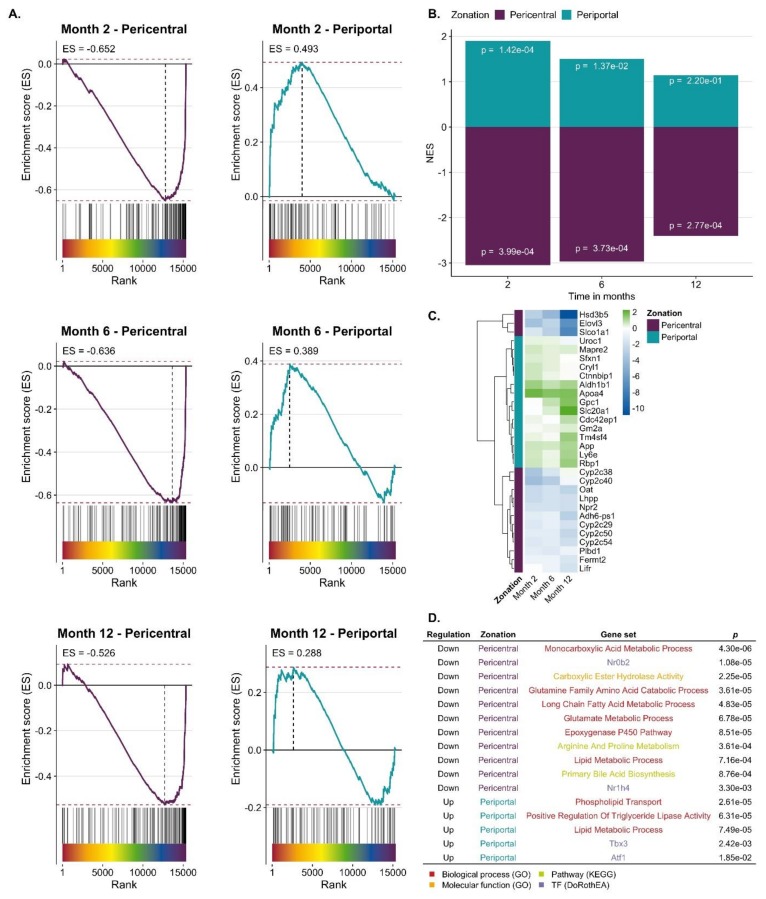
Periportalization of CCl_4_ exposed liver tissue. **A**. Enrichment of pericentral and periportal genes among genes up or downregulated by CCl_4_ exposure. **B**. Summarized results of Gene Set Enrichment Analysis (GSEA) showing normalized enrichment score (NES) and *p*-values. **C**. Leading edge of periportal and pericentral gene set that mainly accounts for the enrichment score of the gene set. The color scheme indicates the logFC. **D**. Selection of GO-terms and TFs that characterize the overlap of CCl_4_ signature and pericentral/periportal gene sets.

CCl_4_-induced expression response in relation to zonated genes. To study a possible zonation of genes up- or downregulated in CCl_4_-induced fibrosis, a consensus list of pericentral and periportal genes was established, containing 136 and 83 genes, respectively (Table S6). To our knowledge, three previous studies identified genes with zonated expression [31,32,33], whose overlap was relatively low (Table S6; Figure S5). Hence, genes were included in the consensus list, when they were identified as pericentral or periportal by at least two of the three published studies (Table S6). Genes were ranked by a gene-level statistic (here moderated t-value provided by limma) indicating the strength of their deregulation in response to CCl_4_ treatment with upregulated genes at the top ranks (left side of the x-axis) and downregulated at the bottom of the list (right) (Figure 3A). Each vertical line on top of the x-axis represents a member of the pericentral or periportal gene set. The y-axis represents the enrichment score (ES), where values higher than zero indicate enrichment of zonated genes among upregulated and values smaller than zero among downregulated genes. Pericentral genes were significantly enriched among downregulated genes at all time points of CCl_4_ treatment (Gene Set Enrichment Analysis (GSEA), *p*-values of 3.99 × 10^−4^ for month 2, 3.73 × 10^−4^ for month 6 and 2.77 × 10^−4^ for month 12). Periportal genes were significantly enriched among upregulated genes only after two and six but not after 12 months of CCl_4_ treatment (GSEA, *p*-values of 1.42 × 10^−4^ for month 2, 0.013 for month 6). (Figure 3B). *P*-values were not adjusted for multiple hypothesis testing as we tested only 2 gene sets per signature. Leading edge analysis identified a set of downregulated pericentral and upregulated periportal genes across all time points that are mainly driving the significant GSEA results (Figure 3C, Table S7). We also characterized the overlap of pericentral/periportal genes and the most responsive genes of CCl_4_ treatment across all time points using over-representation analysis. Analyzing the downregulated pericentral genes, biological processes such as monocarboxylic acid metabolism, epoxygenase P450, and glutamine family catabolic process and the KEGG pathways primary bile acid biosynthesis as well as arginine and proline metabolism were enriched; the transcription factor small heterodimer partner (SHP; synonym: Nr0b2), an interaction partner of HNF4α and LXRα, showed increased activity (Figure 3D). Among the periportal upregulated genes’ GO groups associated with lipid metabolism, triglyceride lipase, and phospholipid transport were enriched. Some hits are listed in Figure 3D and the complete list is available in Table S8. Thus, during CCl_4_-induced liver fibrosis, a complex conglomerate of inflammatory pathways orchestrate downregulation of pericentral and upregulation of periportal genes that further will be referred to as ‘periportalized’ lobular zonation.

**Figure 4:**
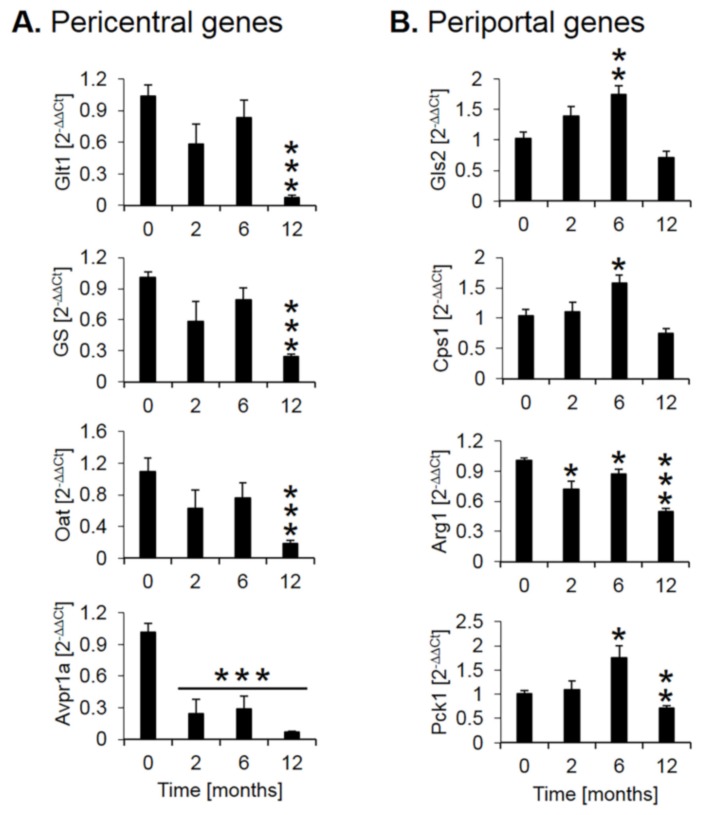
Quantitative real-time polymerase chain reaction (qRT-PCR) confirmation of selected pericentral (**A**) and (**B**) periportal genes. The x-axis represents the time of CCl_4_ treatment, while the y-axis depicts relative RNA expression normalized to controls (0 months). Glt1: glutamate transporter 1; GS: glutamine synthetase; Oat: ornithine aminotransferase; Avpr1a: arginine vasopressin receptor 1A; Gls2: glutaminase 2; Cps1: carbamoyl phosphate synthetase I; Arg1: arginase 1; Pck1: phosphoenolpyruvate carboxykinase 1. The data are means ± standard errors of 6 mice per time point. * *p* < 0.05; ** *p* < 0.01; *** *p* <0.001 compared to the untreated controls (0).

Expression of several genes with a zonated expression pattern was validated by quantitative real-time polymerase chain reaction (qRT-PCR, Figure 4). Analysis of pericentrally expressed genes, solute carrier family 1 member 2 (GLT1), glutamine synthetase (GS), ornithine amino-transferase (Oat), and the vascular/hepatic-type arginine vasopressin receptor (Avpr1a), confirmed a strong downregulation during CCl_4_ treatment, particularly between months 6 and 12 (Figure 4A). In contrast, the periportal genes glutaminase 2 (Gls2), the urea cycle enzyme carbamoyl phosphate synthetase 1 (CPS1) and the gluconeogenesis enzyme phosphoenolpyruvate carboxykinase 1 (PCK1) showed an increase until month 6, followed by a decrease at month 12 (Figure 4B). The urea cycle enzyme arginase 1 (Arg1) showed little change until month six followed by a moderate decrease at month twelve (Figure 4B). Thus, qRT-PCR of selected genes confirmed a strong decrease of the pericentral genes, while the changes of periportal genes are weaker and more complex, characterized by an increase until month 6 and a decrease between months 6 and 12, an observation that will be interpreted in the context of the immunostaining data described below.

**Figure 5:**
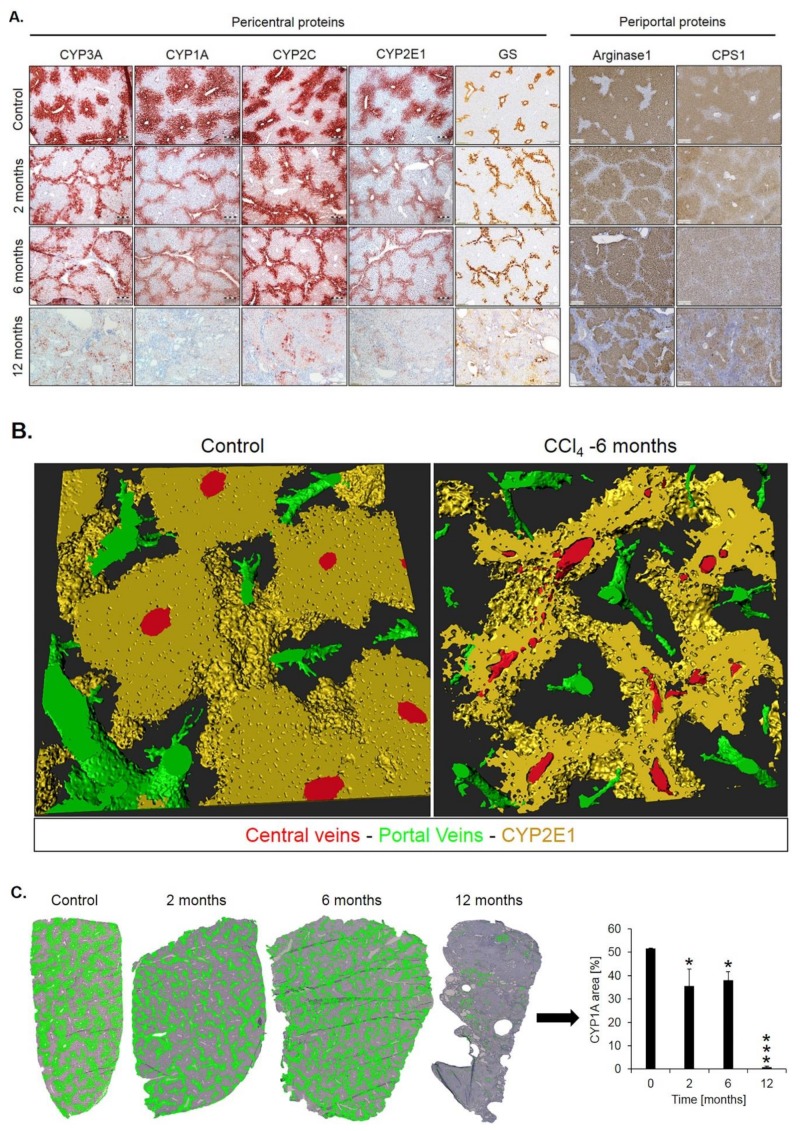

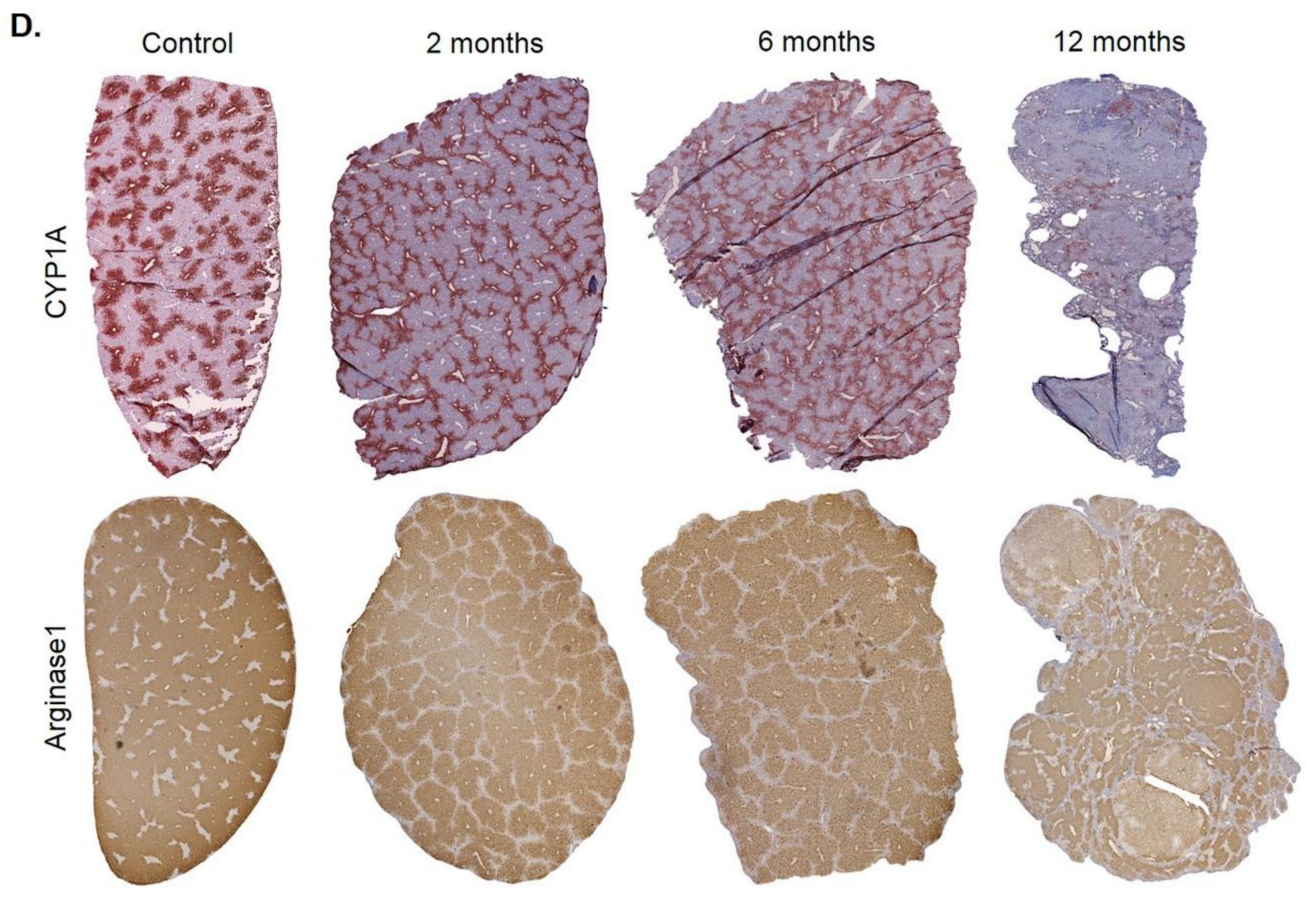
Spatio-temporal analysis of periportalization of selected pericentral and periportal enzymes. (**A**) Immunostaining of the pericentral proteins cytochrome P450 3A, 1A, 2C, 2E and glutamine synthetase (GS) as well as the periportal proteins arginase 1 and carbamoyl phosphate synthetase 1 (CPS1). The left margin indicates the time of treatment with CCl_4_. Scale bars: 200 µm. (**B**) 3D-Reconstructions of CYP1A immunostained liver tissue showing normal pericentral zonation in control (left), and central-to-central bridging at month six of CCl_4_ intoxication. (**C**) Whole slide scans of CYP1A-immunostained liver lobules at 2, 6 and 12 months after CCl_4_ treatment with segmentation (green) and quantification of the fraction of the CYP1A positive area. The data are means ± standard errors of 3 mice per time point. * *p* < 0.05; *** *p* < 0.001 compared to the untreated controls (0). (**D**) Whole slide scans of CYP1A and arginase1 positive liver tissue.

Spatio-temporal analysis of periportalization. Further insight into spatio-temporal changes of zonation was obtained by immunostaining. Similar results were observed for the pericentrally expressed enzymes, cytochrome P450 (CYP) 3A, 1A, 2C, 2E1 and GS (Figure 5A). Compared to controls, the CYP positive areas around central veins became narrower after 2 and 6 months of CCl_4_ administration. However, contacts between CYP2E1 positive areas present in controls (Figure 5B, control; Supplementary Video 1) were maintained even after 6 months of repeated CCl_4_ treatment, giving the impression of central-to-central bridging (Figure 5B, CCl_4_; Supplementary Video 2). Until month 12, CYP immunostaining decreased massively. Similarly, GS showed central-to-central bridging at month two and six, followed by an almost complete loss of expression at month 12 (Figure 5A). Image analysis confirmed the decrease of the immunostained CYP1A1-positive area (Figure 5C). In controls, the periportally expressed urea cycle enzymes, arginase 1 and CPS1 showed a periportal to midzonal staining pattern with a relatively narrow negative pericentral zone (Figure 5A). The vessels in the center of arginase 1 or CPS1 positive regions are portal veins, while the vessels in negative regions represent central veins. During the one-year-period of CCl_4_ administration, fibrotic streets formed between the central veins, which were particularly obvious between months 6 and 12 (Figure 5A). No expression of arginase 1 or CPS1 occurred in the fibrotic streets, while these enzymes were expressed in hepatocytes at similar levels as in control mice, even in the regenerative nodules at month 12. Whole slide scans immunostained for CYP1A and arginase 1 illustrated the narrowing of the pericentral region expressing CYP1A (months 2 and 4), followed by an almost complete loss at month 12 (Figure 5D). Vice versa, arginase1 expression extended into the pericentral region at months 2, 6 and 12 (Figure 5D).

### 3.2. Confirmation of Periportalization in Further Mouse Models of Liver Fibrosis

**Figure 6:**
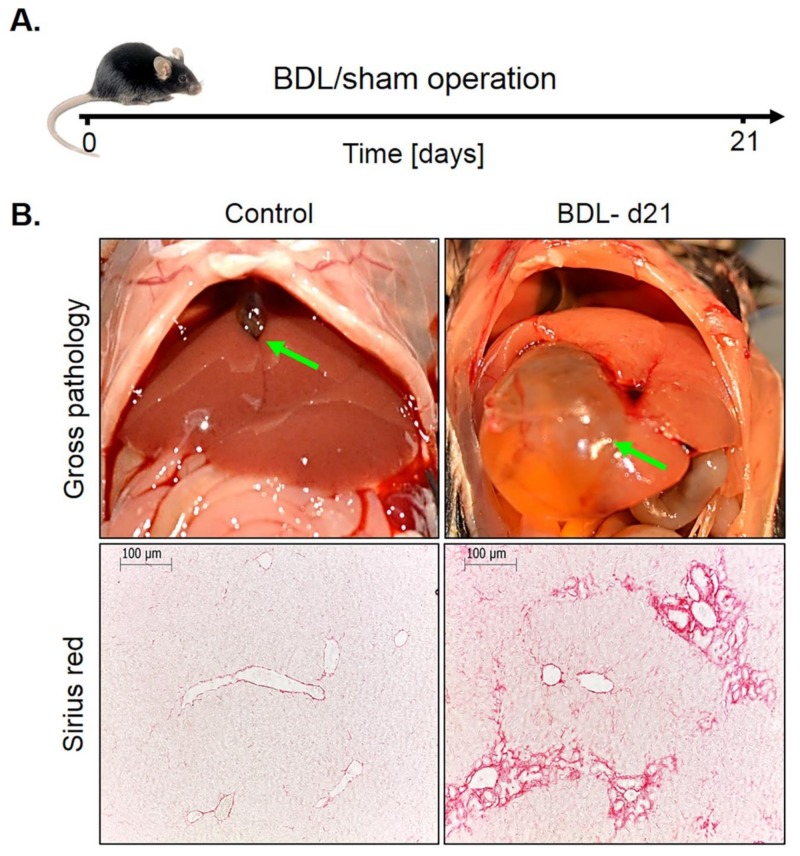
Periportal fibrosis after bile duct ligation (BDL). (**A**) Experimental schedule. (**B**) Macroscopic appearance and visualization of fibrosis by Sirius red staining. Scale bars: 100 µm.

Bile duct ligation (BDL). This mouse model was investigated because it represents a periportal fibrosis model, in contrast to the CCl_4_ model described above, where pericentral fibrosis is induced. Ligation of the common bile duct leads to the formation of bile infarcts due to the rupture of the apical hepatocyte membrane in the acute phase up to day three [22]. In the chronic phase after approximately seven days, the liver adapts to the obstruction of the bile duct, bile infarcts do no longer occur and the infarct regions regenerate. However, a slowly progressing periportal fibrosis occurs in the chronic phase. In the present study, mice at day 21 after BDL were compared to sham-operated controls (Figure 6A). Macroscopically, BDL mice showed a strongly distended gallbladder with transparent, so-called ‘white bile’ (Figure 6B). Histologically, a strong ductular response was observed accompanied by periportal fibrosis (Figure 6B).

**Figure 7:**
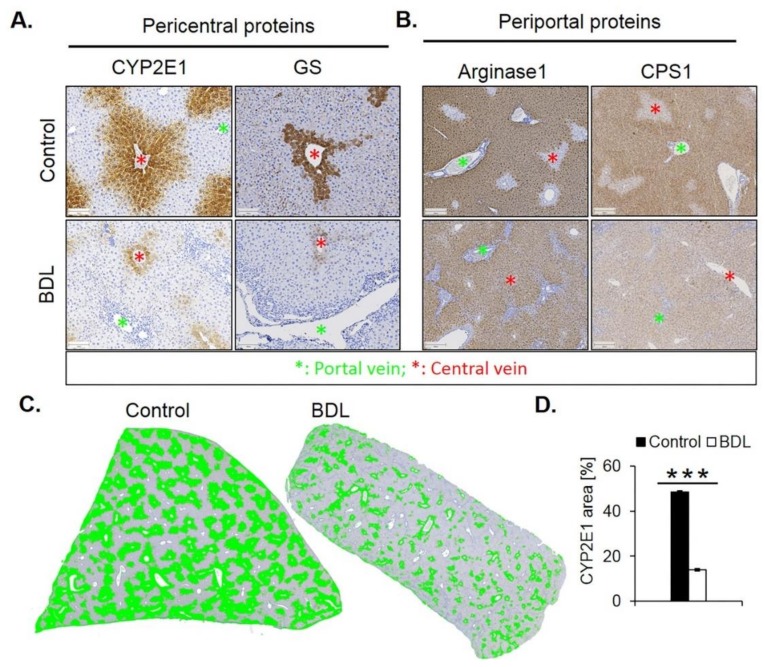
Periportalization of lobular zonation after BDL. (**A**) Immunostaining of the pericentral proteins CYP2E1 and GS. Scale bars: 100 µm. (**B**) Immunostaining of the periportal proteins arginase1 and CPS1. Scale bars: 200 µm. (**C**) CYP2E1-immunostained whole slide scans of liver lobules of BDL mice, and segmentation of the positive area (green). (**D**) Quantification of the fraction of CYP2E1 positive tissue 21 days after BDL and in controls. The data are means ± standard errors of 3 mice per group. *** *p* < 0.001 compared to the sham controls (0).

Immunostaining for CYP2E1 and GS showed a massive decrease 21 days after BDL (Figure 7A). While the CYP2E1 positive area amounted to approximately 50% of the total tissue area in controls, this value fell to only approximately 14% after BDL (Figure 7C,D). Corresponding analysis of the periportal enzymes arginase 1 and CPS1 showed that the negative pericentral regions in controls become positive (‘periportalized’) after BDL (Figure 7B). Whole slide scans immunostained for CYP2E1 and CPS1 confirmed the results shown in Figure 7 (Figure S1). Therefore, BDL associated fibrosis was accompanied by similar changes in zonation as fibrosis induced by chronic administration of CCl_4_. Mdr2^−/−^ mice represent a further model of periportal fibrosis. Similar to the CCl_4_ model and BDL, also eight- and 64-week-old knockout mice showed reduced expression of CYP2E1 (Figure S2).

### 3.3. Functional Consequences of Compromised Zonation: Adaptation to Hepatotoxicants

**Figure 8:**
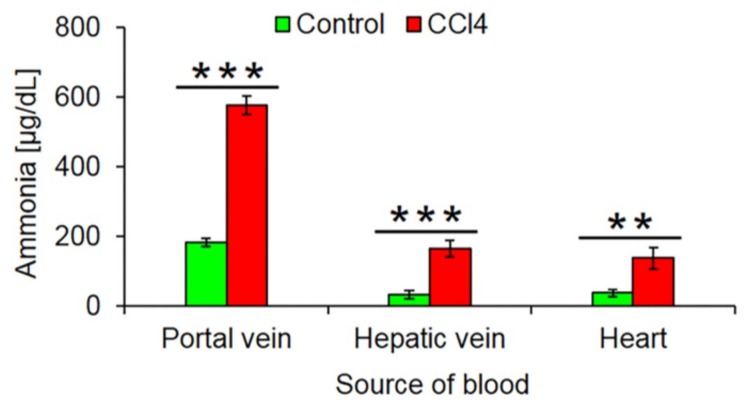
Increased ammonia blood concentrations after 1 year CCl_4_ treatment. The data are means ± standard errors of 6 mice per group. ** *p* < 0.01; *** *p* < 0.001 compared to the corresponding controls (0).

Fibrosis associated disturbed lobular zonation causes several adverse functional consequences. An example is compromised ammonia detoxification. The loss of the fine-tuned interaction of the periportal high capacity (urea cycle) and the pericentral high-affinity (GS) compartments leads to increased ammonia blood concentrations. We compared ammonia concentrations in the portal vein, liver vein and heart blood of mice after 1 year of treatment with CCl_4_ and untreated controls. The much higher concentrations in the hepatic vein of CCl_4_ mice demonstrate the loss of the capacity of the fibrotic liver to reduce ammonia to very low concentrations of <30 µg/dL (Figure 8).

**Figure 9:**
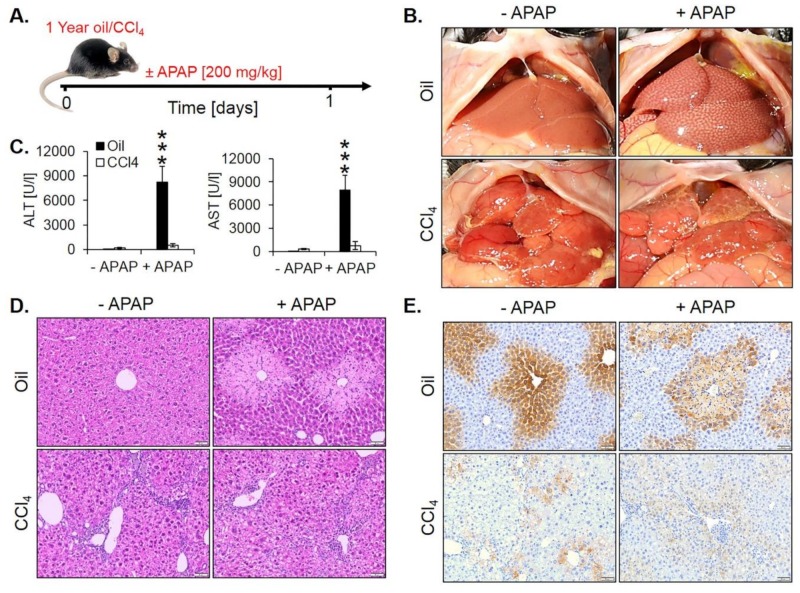
Acetaminophen (APAP) resistance of mice after one year of treatment with CCl_4_. (**A**) Experimental schedule. (**B**) Macroscopic appearance. (**C**) The concentration of liver enzymes in the blood. The data are means ± standard errors of 5 mice per time point. *** *p* < 0.001 compared to the corresponding controls without APAP intoxication. (**D**) H&E-stained tissue. Scale bars: 100 µm. (**E**) CYP2E1 immunostaining. Scale bars: 100 µm.

Moreover, we wondered if the loss of the pericentrally located cytochrome P450 enzymes may lead to resistance against hepatotoxicants that require metabolic activation by these enzymes. To test this hypothesis, we exposed one year CCl_4_-treated fibrotic mice and olive oil controls to a hepatotoxic dose of APAP (200 mg/kg) (Figure 9A). In oil controls, APAP macroscopically caused the characteristic dotted pattern, indicating pericentral necrosis (Figure 9B) that was confirmed histologically (Figure 9D). Interestingly, APAP-induced no visible necrosis in the fibrotic livers after one year of chronic CCl_4_ intoxication (Figure 9B,D, Figure S4). These results were confirmed by analysis of the liver enzymes ALT and AST that increased after APAP administration in oil controls but not in fibrotic livers (Figure 9C). Immunostaining for CYP2E1 illustrated that the CYP2E1 positive pericentral regions were destroyed by APAP (Figure 9E). In contrast, the periportalized fibrotic livers without detectable CYP2E1 expression did not show any necrotic hepatocytes around central veins (Figure 9E). The corresponding H&E-stained whole slide scans are available in Figure S4).

**Figure 10:**
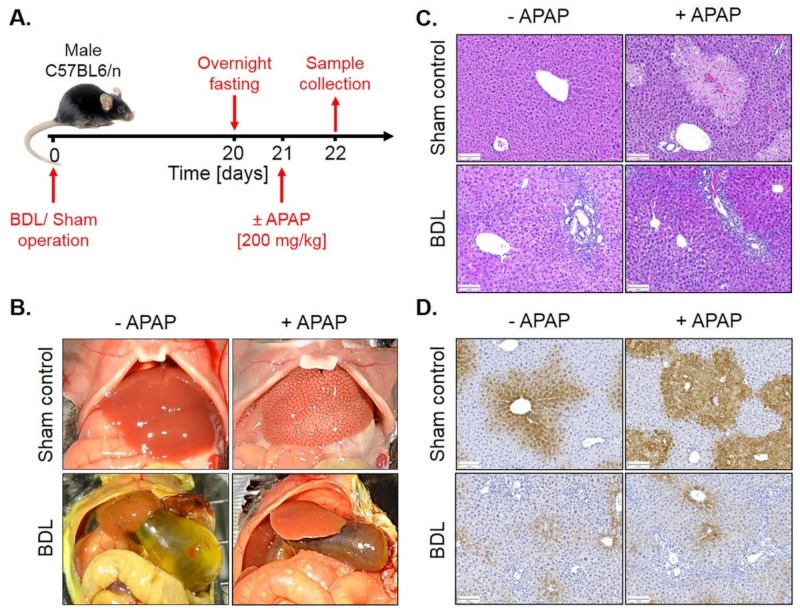
APAP resistance of mice 21 days after BDL. (**A**) Experimental schedule. (**B**) Macroscopic appearance. (**C**) H&E staining. Scale bars: 100 µm. (**D**) CYP2E1 immunostaining. Scale bars: 100 µm.

To analyze whether fibrosis-associated resistance to APAP is a generalizable phenomenon, a similar experiment was performed in mice with BDL-associated fibrosis. For this purpose, BDL mice and sham controls were overnight fasted from the evening of day 20 after surgery and 200 mg/kg APAP were administered on the morning of day 21 to be analyzed 24 h later (Figure 10A). Interestingly, BDL mice were resistant to APAP (Figure 10C,D; Figure S5), similar to the observation made in CCl_4_-induced fibrosis.

## 4. Discussion

Little is known about how liver fibrosis influences hepatic zonation. Here, we show that fibrosis of different etiologies causes ‘periportalization’, which means that the entire liver lobule including the pericentral region adopts a periportal pattern of gene expression (graphical abstract). We first observed this phenomenon in the mouse model of CCl_4_ established here, where severe fibrosis was induced by repeated induction of pericentral liver necrosis over a period of one year. Recently, several genome-wide studies have identified genes that show a preferentially pericentral or periportal expression pattern [31,32,33]. We established a consensus list of periportal and pericentral genes that overlapped in at least two of the three studies. The genome-wide analysis showed that pericentral genes are enriched among the genes downregulated by CCl_4_, while the periportal genes are enriched among the upregulated genes. This phenomenon occurred already at the earliest analyzed time point of two months.

Analysis of selected pericentral genes by immunostaining and qRT-PCR showed that the downregulation of pericentral genes occurred in two phases. Phase 1 until month 6 is characterized by ‘central-to-central bridging’. In this period, the pericentral area with positive immunostaining of e.g., cytochrome P450 enzymes becomes narrower but areas of contact between neighboring pericentral regions are maintained. In this first phase, RNA levels of the analyzed pericentral genes show a statistically significant but moderate, less than 2-fold decrease. Phase 2 occurs between months six and twelve and is characterized by a massive formation of fibrotic streets. In this period, immunostaining of cytochrome P450s and glutamine synthetase is almost completely lost and RNA levels decrease by more than 10-fold.

Immunostaining of the periportal genes arginase 1 and CPS1 shows that they begin to be newly expressed in the pericentral region during CCl_4_ treatment. In controls, they are expressed in periportal and midzonal hepatocytes but not in a narrow region around the central vein. While pericentral proteins retreat, arginase 1 and CPS1 advance towards the central vein until finally all hepatocytes express these periportal genes. The extension of periportal enzymes into the pericentral region is accompanied by a moderate but statistically significant increase in RNA levels (e.g., of CPS1) at months 2 and 6. However, between months 6 and 12, expression of several periportal genes decreases again. This is most likely explained by the massive formation of fibrotic streets that contain myofibroblasts, macrophages and further inflammation-associated cells that do not express (periportal) liver genes. Therefore, periportal liver genes will be diluted by the RNA of the cells forming fibrotic streets. Nevertheless, immunostaining suggests that expression levels, e.g., of arginase 1 and CPS1, do not decrease in the remaining hepatocytes of the regenerative nodules at month 12. Rather, the hepatocyte fraction of total liver mass decreases.

The loss of pericentrally expressed metabolizing enzymes has different consequences. One of them is an increase of ammonia concentrations in the liver vein blood and the systemic circulation. During the passage through the fibrotic, 1-year CCl_4_-treated liver, ammonia concentrations still decrease from ~600 µg/dL concentrations in the portal vein to ~180 µg/dL in the liver vein. However, the destruction of the pericentral high affinity detoxification system by glutamine synthetase [15,16] prevents further reduction of ammonia concentrations to normal levels (<30 µg/dL). The reason for the strongly increased ammonia concentrations in portal vein blood (~600 µg/dL) may be due to increased intestinal ammonia production from urea. Because of chronic liver damage mice develop secondary kidney damage [22] with increased blood concentrations of urea. Because of the leaky gut-blood barrier in chronic liver damage, urea reaches bacterial ureases in the intestinal lumen that generate ammonia, which is absorbed into intestinal capillaries and drains into the portal vein [44,45,46]. However, the latter mechanism still requires experimental validation. The data clearly show that ‘periportalization’ of liver lobules causes loss of detoxifying functions. The increase in ammonia or urea concentrations to the observed levels will lead to long-term consequences but is not immediately life-threatening. This is in contrast to acute intoxication by hepatotoxicants that may be lethal. In this context, the loss of pericentrally expressed metabolizing enzymes can be interpreted as an adaptive response to chronic CCl_4_ exposure. CCl_4_ is metabolically activated by cytochrome P450 2E1 and further CYPs to form the reactive trichloromethyl free radical and the trichloromethyl peroxyl radical [2]. Therefore, the downregulation of cytochrome P450 enzymes is an efficient strategy to survive repeated exposure to toxins that require metabolic activation. This leads to the question as to why also mouse models of periportal fibrosis cause downregulation of pericentral genes. In the present study, we used BDL that is known to preferentially cause periportal liver damage and fibrosis [47,48]. Also, mdr2^−/−^ mice are a model of periportal fibrosis [49]. At first glance, it may be difficult to understand why the downregulation of pericentral genes should offer a survival advantage to BDL and mdr2^−/−^ mice. In this context, co-evolution between plants and herbivores should be considered [50]. Plants responded to herbivory by formation of plant toxins, many of them metabolically activated by pericentral liver enzymes, such as pyrrolizidine alkaloids and mycotoxins [51]. Herbivores responded by several evolutionary strategies, particularly by novel detoxifying enzymes. Also the periportalization of liver lobules under toxic stress could be interpreted as an adaptive process in animal–plant warfare. Periportalization seems to represent a stereotypical response to different types of inflammatory stress, possibly because the liver lacks the ability to activate distinct adaptive zonation programs for pericentral and periportal damage. This corresponds to previous studies, demonstrating that different types of acute and chronic inflammatory stimuli activate the same gene regulatory networks, whereby upregulation of inflammatory genes occurs simultaneously to the downregulation of metabolic genes [52]; interestingly, both inflammatory and metabolic genes are controlled by the same upstream mechanisms [52]. A strength of the present study is that six individual mice were included for each condition of the time-resolved analysis. This allowed sufficient statistical power to demonstrate the significance of the process of periportalization identified here.

In conclusion, liver fibrosis leads to periportalization of liver lobules. Periportalization occurs as a common response to pericentral and also periportal damage. It allows the liver to adapt to the repeated exposure to hepatotoxic compounds that require metabolic activation by pericentrally expressed enzymes.

## Figures and Tables

**Table 1:** Antibodies and staining conditions.

Target	Tissue Section	Primary Antibody		Secondary Antibody	
		Antibody	Dilution	Antibody	Dilution
CYP3A	Frozen	Rabbit anti-CYP3A1	1:250	Swine anti-rabbit	1:20
CYP1A	Frozen	Rat anti-CYP1A2	1:500	Rabbit anti-rat IgG	1:1000
CYP2C	Frozen	Rat anti-CYP2C6	1:250	Rabbit anti-rat IgG	1:1000
CYP2E1	Frozen/ FFPE	Rabbit anti-CYP2E1	1:100	Swine anti-rabbit	1:20
GS	FFPE	Mouse anti-GS	1:1000	anti-mouse	1:500
Arginase1	FFPE	Anti-arginase-1 antibody, rabbit monoclonal	1:500	Swine anti-rabbit	1:20
CPS1	FFPE	Anti-CPS1 antibody—liver mitochondrial marker	1:500	Swine anti-rabbit	1:20

## Data Availability

The code to perform the presented RNA-seq study is written in R and is freely available on GitHub: https://github.com/saezlab/LiverPeriportalization [53].

## Supplementary Materials

The following are available online at https://www.mdpi.com/2073-4409/8/12/1556/s1: **Supplemental figures.** Figure S1. Immunostaining of CYP2E1 and CPS1 in whole slide scans after BDL. Figure S2. Sirius red and CYP2E1 staining of 8 and 64 week-old mdr2^−/−^ mice and 64 week-old wild type (WT) mice. Figure S3. H&E staining of livers (whole slide scans) after 1-year exposure to olive oil and CCl_4_. Figure S4. H&E staining of livers (whole slide scans) 21 days after BDL compared to sham-operated controls. Figure S5. Overlap of pericentral and periportal genes between three different studies. **Supplemental tables.** Table S1. Significantly (abs(logFC) ≥ 1.5 and FDR ≤ 0.05) up and downregulated genes after 2, 6 and 12 months of treatment of the mice with 1 g/kg CCl_4_. Table S2. Characterization of CCl_4_ induced expression response by PROGENy pathway analysis. Table S3. Transcription factor activities of genes differentially expressed after CCl_4_ exposure. The column confidence is an empirical classification indicating how confident we are about the TF activity prediction based on the confidence of its regulon. The confidence class range from A–E with being A the highest confident class. Table S4. Analysis of GO group enrichment based on genes differentially expressed after CCl_4_ exposure. Only those GO terms are shown that were significantly enriched (FDR ≤ 0.05) in at least one-time point. Table S5. Significantly (abs(logFC) ≥ 1.5 and FDR ≤ 0.05) up and downregulated genes after 2, 6 and 12 months of treatment of the mice with olive oil. Table S6. List of zonated pericentral and periportal genes. A. Consensus list including genes that were identified as zonated in at least two of the three studies Braeuning et al., 2006; Saito et al., 2013; Halpern et al., 2017. B. Zonated genes in Braeuning et al., 2006; C. Zonated genes in Saito et al., 2013; D. Zonated genes in Halpern et al., 2017. Table S7. Table of leading-edge genes for pericentral and periportal gene set members that mainly account for the corresponding gene set enrichment score. Table S8. Significantly over-represented gene sets (p-value ≤ 0.05) that characterize the overlap of the CCl_4_ signature and pericentral/periportal gene set, respectively. **Supplemental Videos.** Supplemental video 1. 3D reconstructions of control mouse liver immunostained for CYP2E1. Supplemental video 2. 3D reconstructions of fibrotic mouse liver on month 6 of repeated CCl_4_ intoxication immunostained for CYP2E1.

## Abbreviations

APAP	acetaminophen
ALT	alanine transaminase
AST	aspartate transaminase
i.p.	intraperitoneal
BDL	bile duct ligation
PBS	phosphate-buffered saline
CCl4	carbon tetrachloride
CYP450	cytochrome P450
CPS1	carbamoyl phosphate synthase
FFPE	formalin-fixed paraffin embedded
GS	glutamine synthetase
Mdr2	multidrug resistance gene 2
RNA-seq	RNA sequencing
TGFβ	Transforming growth factor beta
NFκB	nuclear factor kappa-light-chain-enhancer of activated B cells
TNFα	Tumor necrosis factor alpha
TCA-cycle	tricarboxylic acid cycle
GAPDH	Glyceraldehyde-3-phosphate dehydrogenase
UMI	unique molecular identifier
GSEA	Gene Set Enrichment Analysis
MGI	Mouse Genome Informatics
