# Supplementary figures and images for: Influence of Liver Fibrosis on Lobular Zonation
Source: Cells. 2019 Dec 2;8(12):1556. doi: 10.3390/cells8121556 (PMC6953125; doi:10.3390/cells8121556)

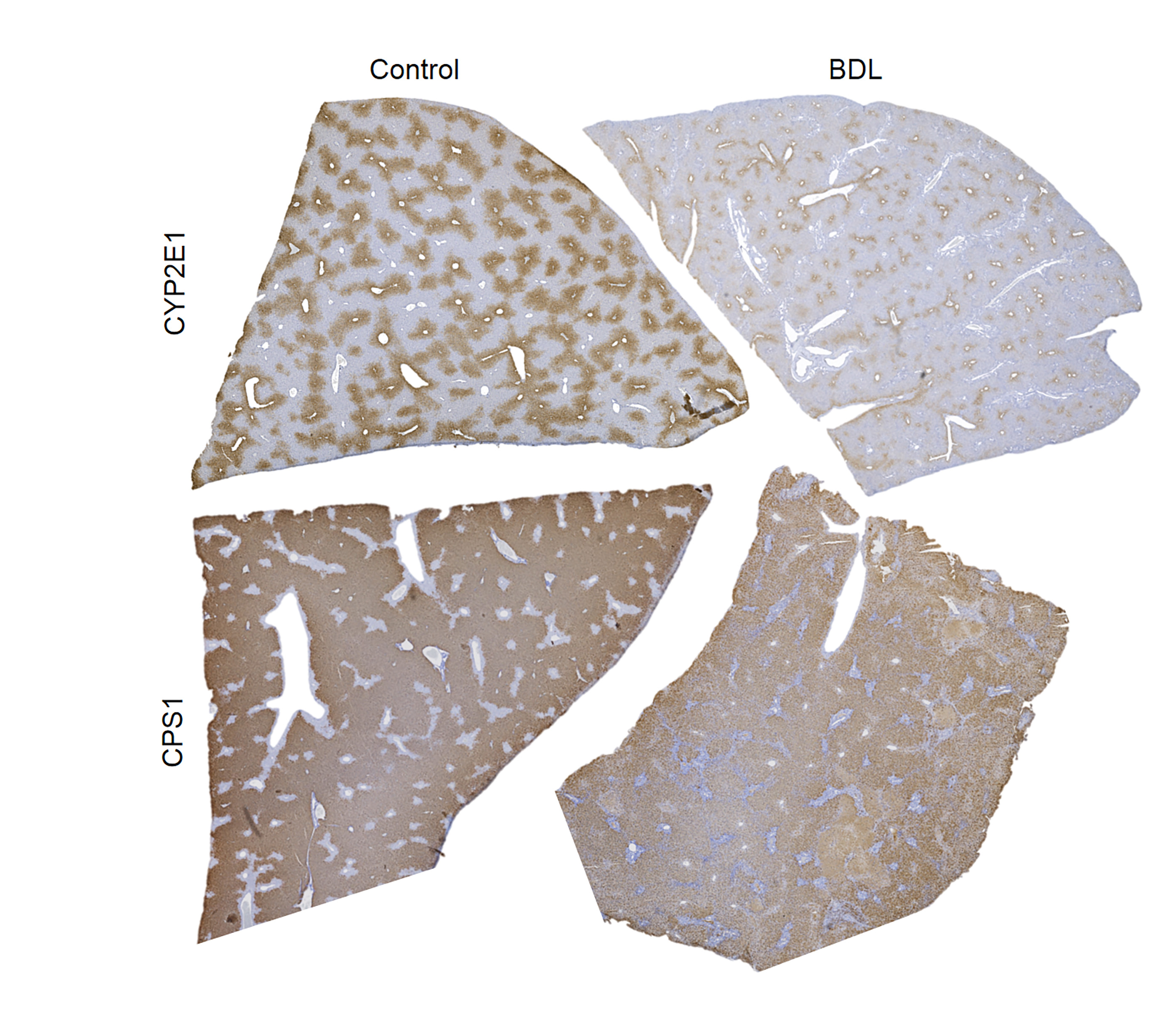

Supplement: Supplementary file 1 [file cells-08-01556-s001.zip › Final Supplementary data/Supplemental figures/supplemental figure 1.jpg]

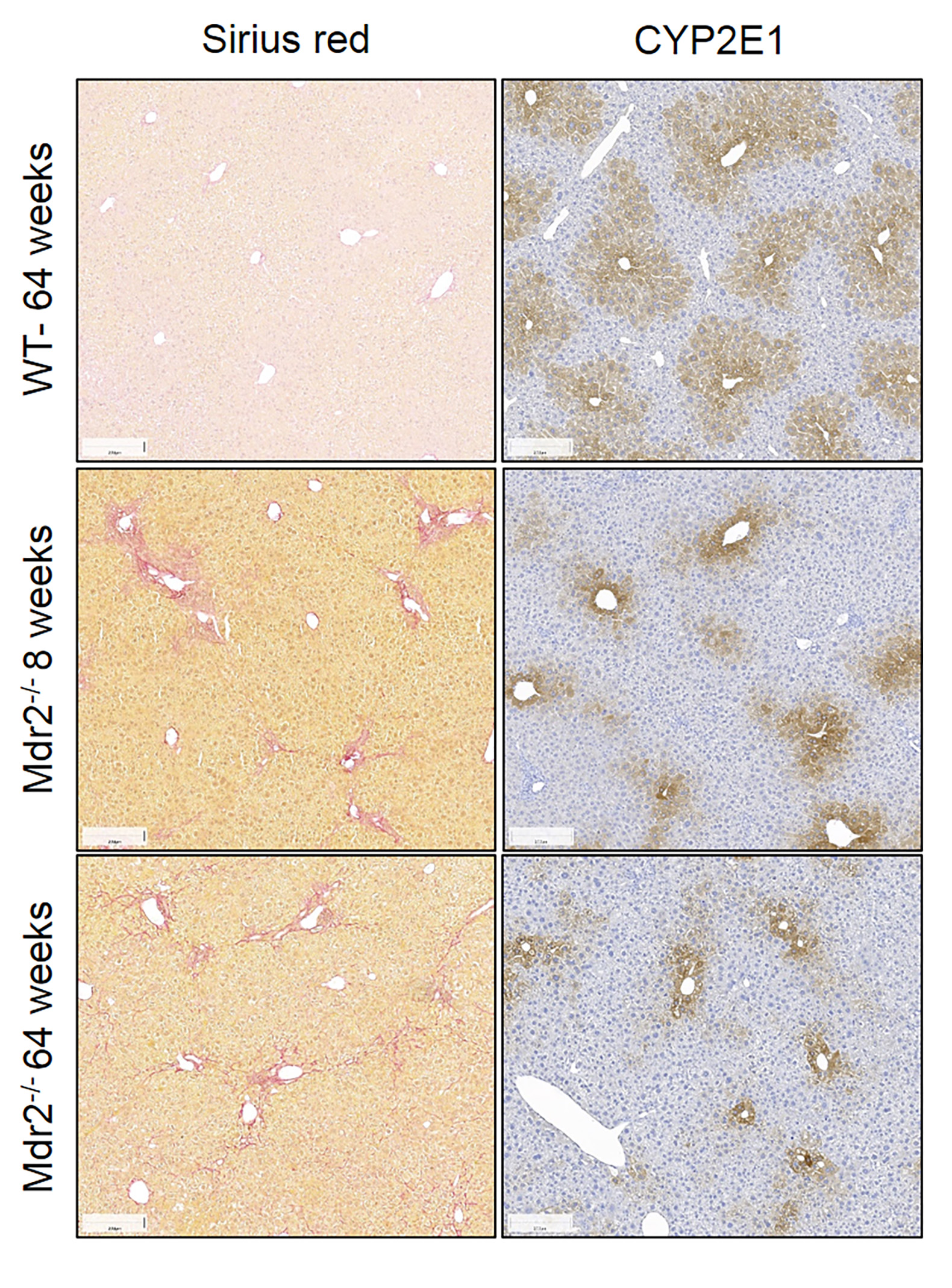

Supplement: Supplementary file 1 [file cells-08-01556-s001.zip › Final Supplementary data/Supplemental figures/supplemental figure 2.jpg]

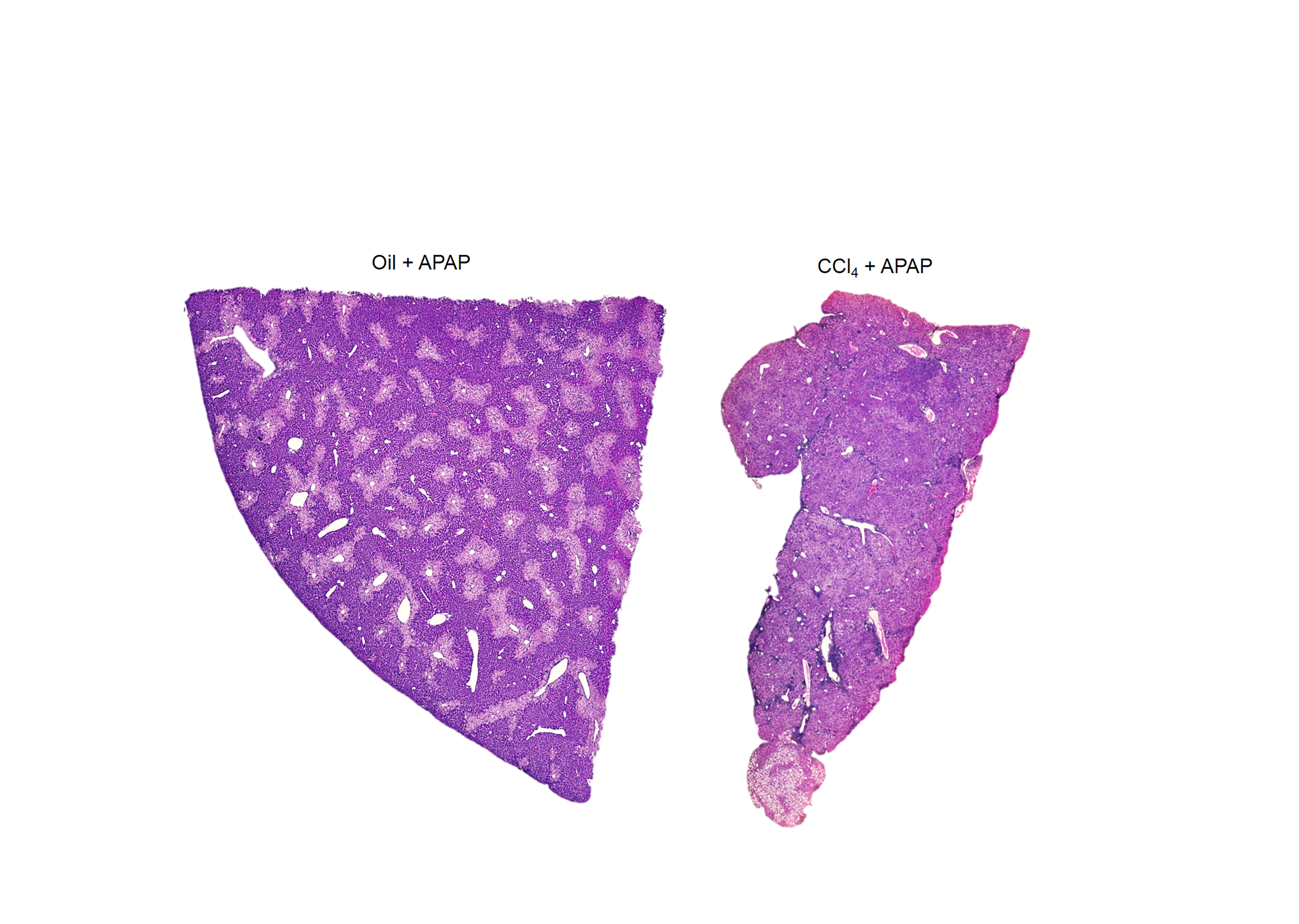

Supplement: Supplementary file 1 [file cells-08-01556-s001.zip › Final Supplementary data/Supplemental figures/supplemental figure 3.jpg]

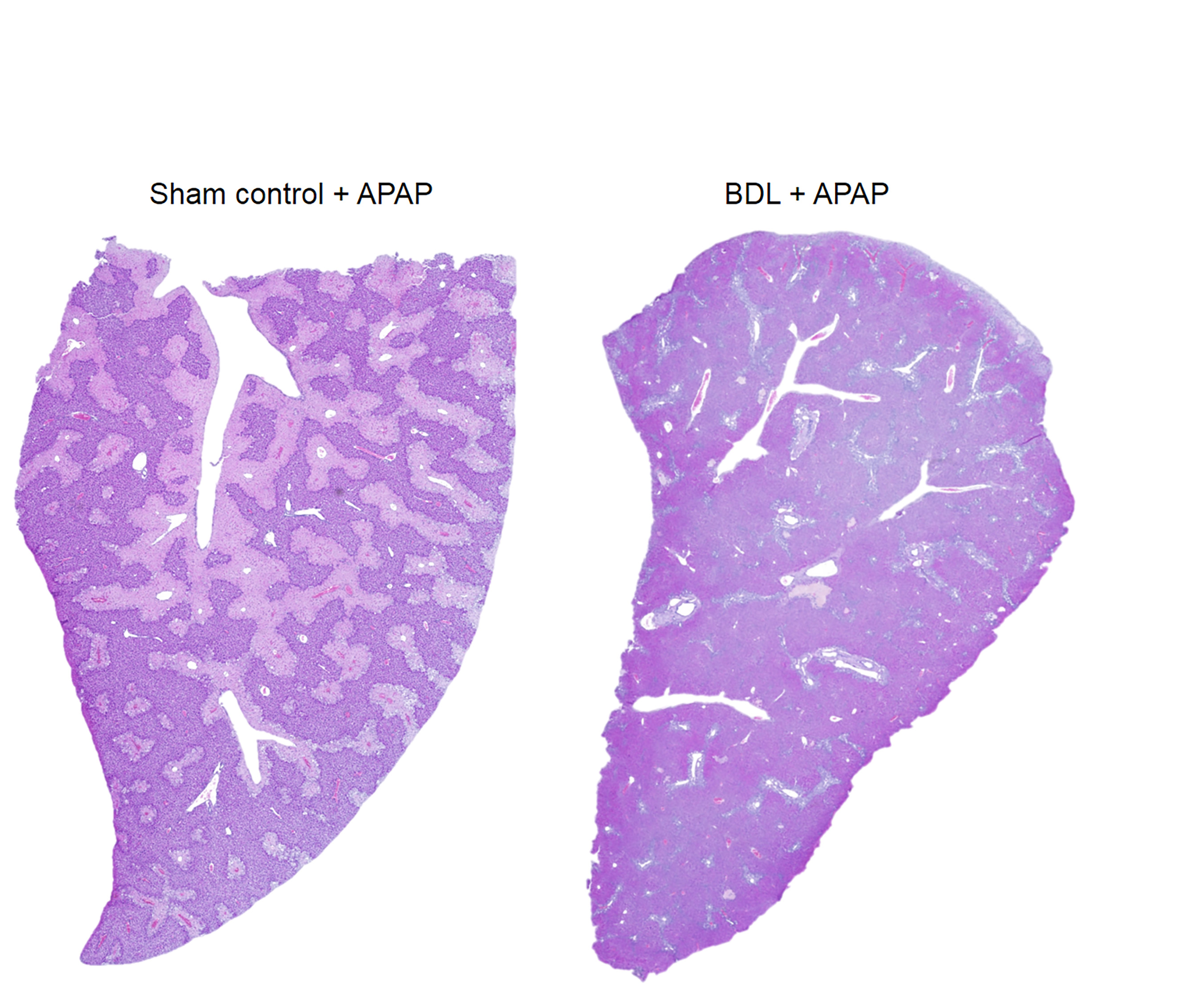

Supplement: Supplementary file 1 [file cells-08-01556-s001.zip › Final Supplementary data/Supplemental figures/supplemental figure 4.jpg]

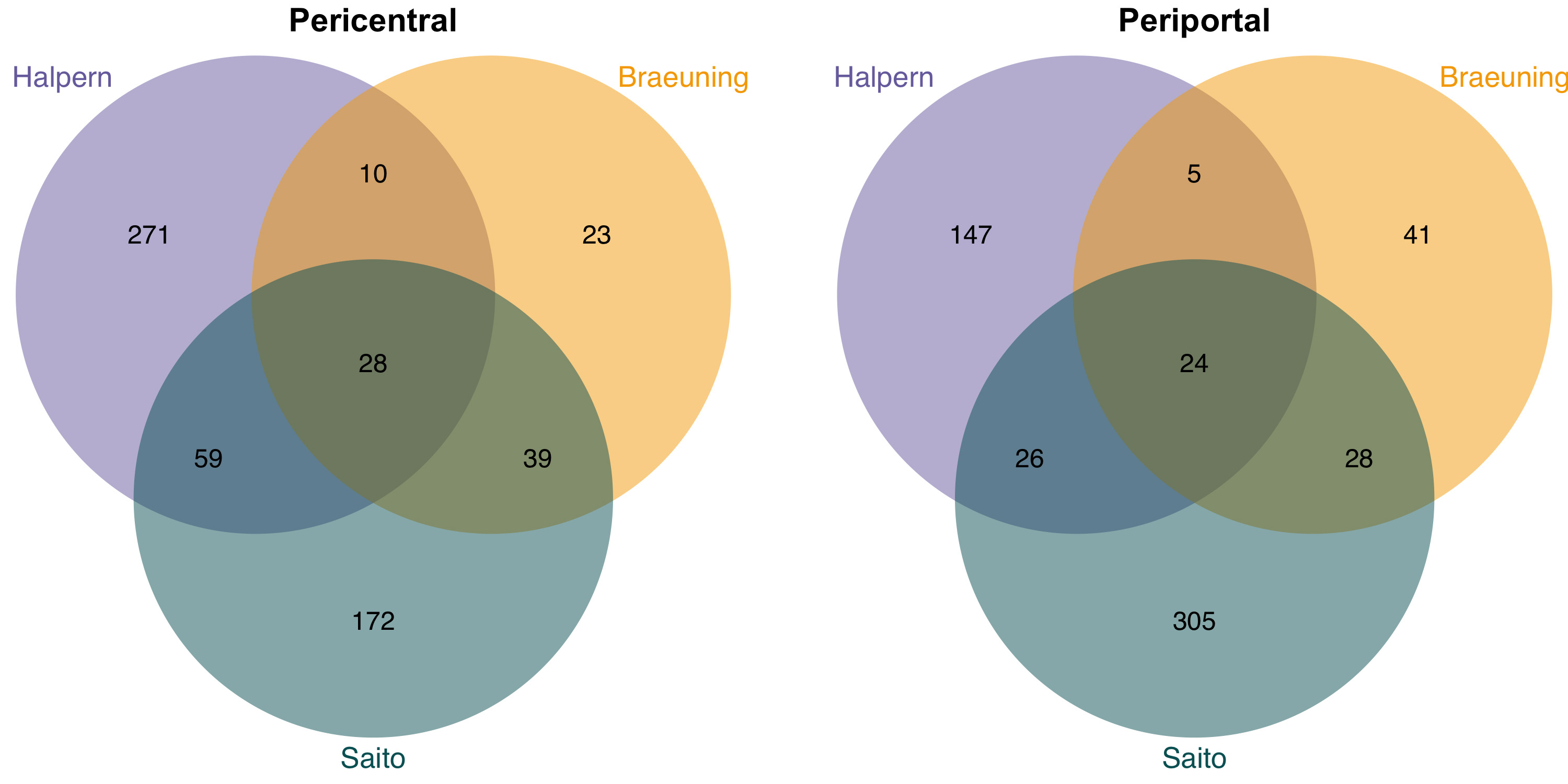

Supplement: Supplementary file 1 [file cells-08-01556-s001.zip › Final Supplementary data/Supplemental figures/supplemental figure 5.jpg]
